# Oxygen: A Fundamental Property Regulating Pelagic Ecosystem Structure in the Coastal Southeastern Tropical Pacific

**DOI:** 10.1371/journal.pone.0029558

**Published:** 2011-12-28

**Authors:** Arnaud Bertrand, Alexis Chaigneau, Salvador Peraltilla, Jesus Ledesma, Michelle Graco, Florian Monetti, Francisco P. Chavez

**Affiliations:** 1 Institut de Recherche pour le Développement (IRD), UMR212 EME IFEREMER/IRD/UM2, Sète, France; 2 Instituto del Mar del Perú, Esquina Gamarra y Gral, Valle s/n, Callao, Lima, Peru; 3 IRD, UMR LEGOS, Toulouse, France; 4 IRD, IPSL/LOCEAN, UPMC/CNRS/IRD/MNHN, Paris, France; 5 Monterey Bay Aquarium Research Institute, Moss Landing, California, United States of America; Utrecht University, The Netherlands

## Abstract

**Background:**

In the southeastern tropical Pacific anchovy (*Engraulis ringens*) and sardine (*Sardinops sagax*) abundance have recently fluctuated on multidecadal scales and food and temperature have been proposed as the key parameters explaining these changes. However, ecological and paleoecological studies, and the fact that anchovies and sardines are favored differently in other regions, raise questions about the role of temperature. Here we investigate the role of oxygen in structuring fish populations in the Peruvian upwelling ecosystem that has evolved over anoxic conditions and is one of the world's most productive ecosystems in terms of forage fish. This study is particularly relevant given that the distribution of oxygen in the ocean is changing with uncertain consequences.

**Methodology/Principal Findings:**

A comprehensive data set is used to show how oxygen concentration and oxycline depth affect the abundance and distribution of pelagic fish. We show that the effects of oxygen on anchovy and sardine are opposite. Anchovy flourishes under relatively low oxygen conditions while sardine avoid periods/areas with low oxygen concentration and restricted habitat. Oxygen consumption, trophic structure and habitat compression play a fundamental role in fish dynamics in this important ecosystem.

**Conclusions/Significance:**

For the ocean off Peru we suggest that a key process, the need to breathe, has been neglected previously. Inclusion of this missing piece allows the development of a comprehensive conceptual model of pelagic fish populations and change in an ocean ecosystem impacted by low oxygen. Should current trends in oxygen in the ocean continue similar effects may be evident in other coastal upwelling ecosystems.

## Introduction

It has been known for some time that oxygen plays a strong role in regulating the coastal benthic communities off the southeastern tropical Pacific and other low oxygen environments [1]. Oxygen levels over the continental shelf off Peru are on average anoxic and only anaerobic filamentous bacteria can survive in the benthos [1]–[4]. Interannually, El Niño oxygenates these benthic habitats that are then colonized by communities that are more typical of other oxygenic environments [1], [4]. Much less is known about the role of oxygen in regulating pelagic ecosystems, in particular metazoan organisms. Given recent reports that oxygen in the world oceans might be decreasing [5]–[7] it is critical that we understand the role of oxygen in structuring pelagic ocean ecosystems. To gain some insights we look at an ecosystem, the coastal southeastern tropical Pacific, which has been experiencing very low, anoxic oxygen conditions for the last few centuries [8]. This ecosystem is also the most productive in the world in terms of fish and one that is subject to large interannual to multi-decadal changes in ocean physics and ecosystem productivity and structure [9].

Eastern boundary upwelling systems (EBUS), i.e., Benguela, California, Canary and the Humboldt Current systems support two heavily exploited fish resources, anchovy (the genus *Engraulis*) and sardine (the genera *Sardinops* and *Sardina*). Observations over the past several decades show that these species varied on multi-decadal time scales and often in an out of phase relationship [10]–[15] and the literature describes these periods of varying abundances as anchovy or sardine ‘regimes’ [10], [13].

A number of hypotheses have been suggested for these fluctuations in anchovies and sardines including sea-surface temperature (SST), food availability and prey size spectra (e.g. [12] and [15]). In EBUS anchovy and sardine are associated with relatively cold and warm conditions, respectively [10], [12], [16]. Cool conditions are accompanied by more nutrients in the euphotic layer and the development of large phytoplankton (e.g. chain-forming centric diatoms) and zooplankton (e.g. large copepods and euphausiids) [17]. Such conditions are predicted to favor anchovy that feed primarily by direct biting on large zooplankton [18]–[19]. In contrast warmer conditions are associated with lower nutrient concentration in the near-surface layer that favor the development of smaller phytoplankton and zooplankton [17] that sardines can more efficiently filter-feed [15].

This relatively simple scheme might indeed explain the anchovy/sardine fluctuations observed during the last decades in the southeastern tropical Pacific off Peru. Historical data show that anchovy (*Engraulis ringens*) was particularly abundant during the relatively cold periods that occurred during the 1960s to the early 1970s, was replaced by the sardine (*Sardinops sagax*) during the warmer years after, which in turn collapsed during the late 1990s and has been virtually absent since the early 2000s, with a return to anchovy abundance [13]. However, the sardine is tolerant of a variety of oceanographic conditions including large temperature ranges (∼9–25°C), and can associate with diverse water masses [20]–[23]. Temperature alone cannot therefore explain the fluctuations in sardine recruitment and distribution [20], [21], [24], [25]. Furthermore, it has also been demonstrated that the sardine can forage on macrozooplankton, with euphausiids constituting more than one third of sardine prey carbon content [26]. If sardine can physiologically tolerate relatively low temperatures and forage on both the large and small sizes of zooplankton, why did it collapse in the late 1990s when (large) prey were abundant and temperature was well within its range of tolerance? Sardine overfishing during the late 1990s was probably an important factor [11], [21], [27] but probably not the unique one. Indeed, analysis of scale or bone remains in sediment cores, show decadal and multi-decadal fluctuations of anchovy and sardine biomass before the development of industrial fishing [8]. Also, during the last two centuries, only two multi-decadal periods of expansion of sardine occurred off Peru [8]. Moreover, periods of anchovy and sardine coexistence have been observed in the past [8], [28]–[29] and in other EBUS [25], [29]. Thus, there may be other parameters than sea-surface temperature, food availability and prey size-spectra that explain pelagic fish fluctuations off Peru.

Here we propose that the need to breathe may be the critical property determining ecosystem structure in the productive waters off Peru. Effectively, fish need sufficient amounts of both food and oxygen, but the latter might be more difficult to obtain than the former [30]. EBUS are associated with oxygen minimum zones (OMZs) where subsurface layers are low in dissolved oxygen (DO). These OMZs, have notable effects on the distribution of marine organisms [1], [7], [31]–[36]. Coastal upwelling then transports low and unsaturated concentrations of DO to the surface [37]. Reduced oxygen can impact fish in at least two ways: (i) by directly affecting fish breathing in the layer where they are distributed, (ii) by compressing their vertical habitat.

We propose that the spatial and temporal dynamics of near-surface DO, percentage of dissolved-oxygen saturation (DO_sat_), and oxycline depth (Z_2 mL/L_) and the impact and regulate anchovy, sardine distribution and abundance in the coastal southeastern tropical Pacific. The impact of oxygen also depends on the food web structure and fish energetic need to efficiently forage. We use a comprehensive fisheries oceanography dataset, collected by the Instituto del Mar del Peru (IMARPE), to investigate the effect of oxygen on fish at three scales: (i) decadally through fluctuations in fish landings (1964–2008), using virtual population analysis (VPA) biomass (1964–2006) and biomass estimated acoustically (1983–2008); (ii) cross-shore fish biomass distributions; and (iii) the small scale patterns of fish acoustic biomass (1983–2005). The results illustrate the critical role of oxygen on fish distribution from the perspective of the two primary fish survival requirements, the need to eat and the need to breathe. We show that oxygen fills a current gap in our understanding of pelagic fish fluctuations and explains the recent sardine collapse leading us to propose a conceptual model of spatiotemporal variations in pelagic fish populations off Peru at multiple scales.

## Results

### Temporal patterns

**Figure 1:**
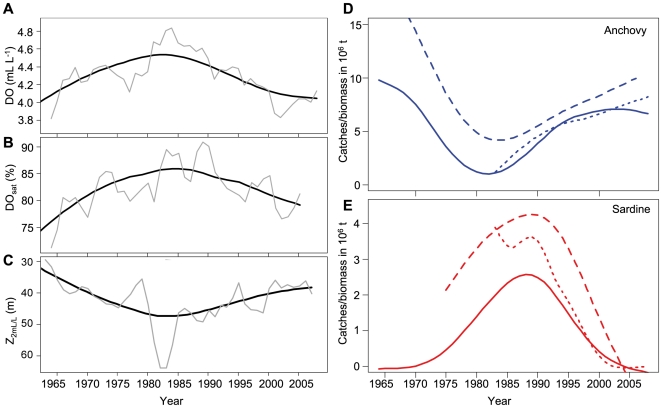
Time series (1964–2009) of oxygen and fish. Temporal variations of DO in mL L^−1^ (black dotted line) (**A**), DO_sat_ in % (black dashed line) (**B**), Z_2 mL/L_, in m (black solid line). Black solid line show the smoothed time series; gray solid line show the 4-years moving average. **C**. anchovy catches (blue solid line), biomass by VPA (blue dashed line) and acoustic biomass (blue dotted line). **D**. Temporal variations of sardine catches (red solid line), biomass by VPA (red dashed line) and acoustic biomass (red dotted line).

**Figure 2:**
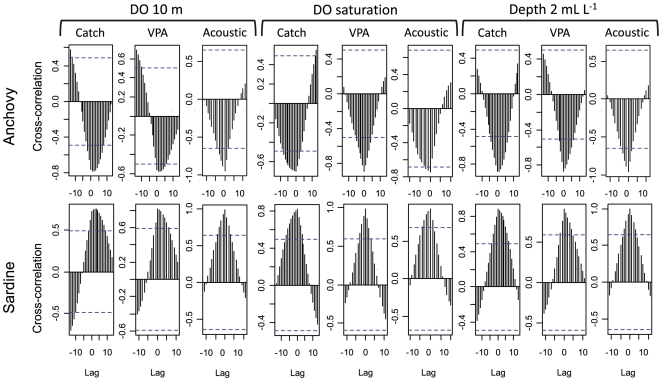
Time-lagged cross correlations of oxygen and fish. Time-lagged cross-correlations in year between smoothed times series of anchovy and sardine catches and biomass by VPA and acoustic biomass and DO, DO_sat_ and Z_2 mL/L_. Values above (below, respectively) the top (bottom) dotted lines are significant at p = 0.001.

DO, DO_sat_ and Z_2 mL/L_ presented similar decadal patterns with low values in the 1960s followed by an increase during the 1970s, reaching a maximum during the 1980s, followed by a decrease during the 1990s–2000s (Fig. 1A,B,C). Anchovy catch and biomass (estimated by both VPA and acoustic) were higher during periods of lower DO and DO_sat_ and shallower Z_2 mL/L_ (Fig. 1D). Anchovy and oxygen showed significant (*P*<0.001) negative correlation (Fig. 2; see Figure S1 for cross correlation preformed on unsmoothed series). The peak in cross-correlation occurred at a lag of 0 years, except for anchovy catches and VPA-estimated biomass and DO where it occurred at a lag of 1 year (Fig. 2). In contrast, sardine catch and biomass (Fig. 1E) were in-phase with the oxygen time series; sardine being abundant when DO and DO_sat_ were higher than ∼4.3 mL L^−1^ and ∼80%, respectively and Z_2 mL/L_ deeper than ∼40 m (Fig. 1). Note at this scale that the range of variation of oxygen variables is lower than at other scales, which is logical since we are averaging data over a large area (both along-shore and cross-shore). The in-phase relationships were highly significant (*P<0.001*; Figs. 2 and S1) and cross-correlation analyses peaked at a lag of 0 year in all cases, except between sardine caches and DO where the peak occurred at +2 years.

### Cross-shore patterns

**Figure 3:**
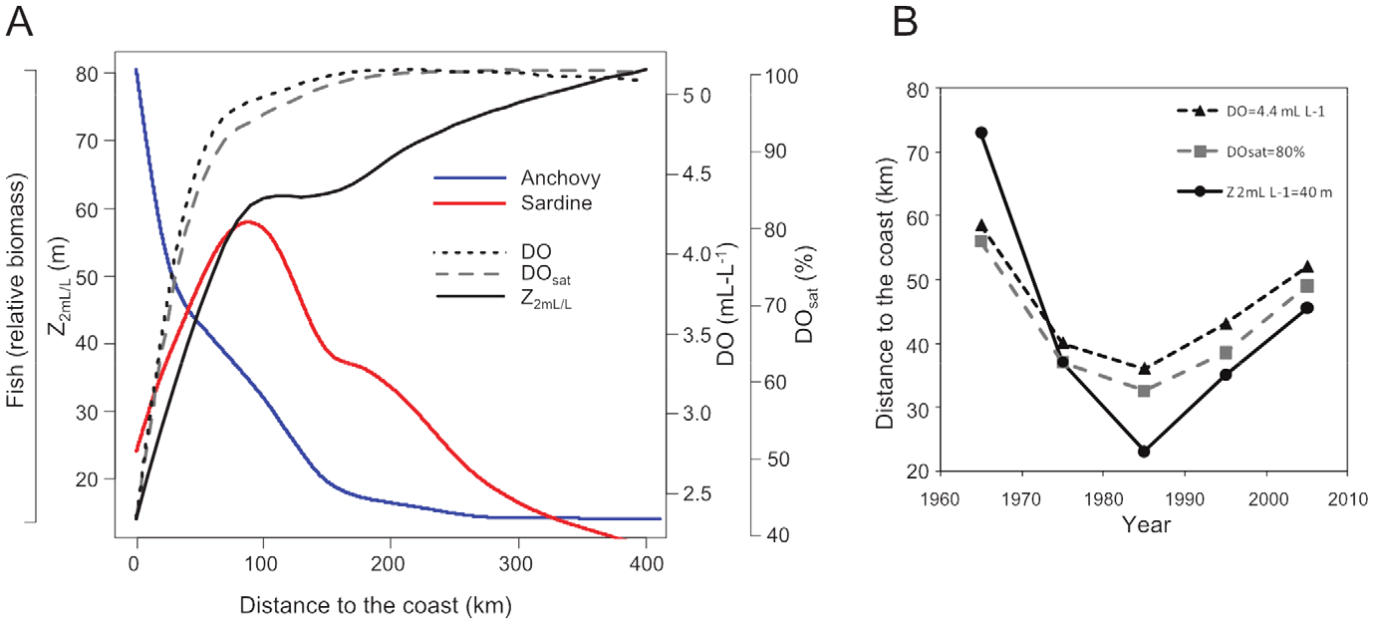
Cross-shore profiles of oxygen and fish biomass. **A**. Mean cross-shore profiles of DO in mL L^−1^ (black dotted line), DO_sat_ in % (grey dashed line), Z_2mL/L_ in m (black solid line), and the acoustic-estimated biomass of anchovy (blue solid line) and sardine (red solid line). **B**. Decadal evolution, from the 1960s to the 2000s, of the distance from the coast of the DO, DO_sat_ and Z_2mL/L_ isovalues equal to 4.4 mL L^−1^, 80% and 40 m, respectively.

The mean (1983–2005) cross-shore evolution of DO, DO_sat_, Z_2 mL/L_ and pelagic fish relative biomass in the study region is shown in Figure 3A. The upwelling of highly remineralized, suboxic water is evident close to the coast as relatively low DO (∼2.5 mL L^−1^) and DO_sat_ (∼40%), and shallow oxycline depth (Z_2 mL/L_ = ∼15 m). DO and DO_sat_ show a sharp linear increase in the offshore domain reaching values of ∼5 mL L^−1^ and ∼95%, respectively, at 100 km from the coast. DO shows a near-constant value of 5.1 mL L^−1^, corresponding to the 100% saturation level, in offshore waters (>150 km from the coast). As with other studies (e.g. [38]), Z_2 mL/L_ rapidly deepened from ∼15 m at the coast to ∼60 m at 100 km, then slowly deepened to 80 m at 400 km from the coast.

Anchovy abundance was inversely correlated with oxygen showing a maximum close to the coast in the poorly-oxygenated near-surface water, decreasing sharply to a distance of ∼30 km and then gently after (Fig. 3A). Over the study period the anchovy distribution was typically restricted to within 150 km from the coast and 50% of the anchovy biomass was associated with DO values lower than ∼3.5 mL L^−1^ which corresponds to saturation levels <65% and an oxycline depth shallower than ∼30 m. In contrast, sardine biomass displayed a minimum next to the coast and increased offshore reaching a maximum at ∼80–100 km from the coast where near-surface water masses are relatively well-oxygenated, with DO and DO_sat_ values of ∼4.9 mL L^−1^ and ∼90% respectively, and the oxycline is deeper than ∼60 m. Further from shore sardine biomass progressively decreased. Sardine were preferentially (>80% of their cumulative biomass) distributed where DO and DO_sat_ were higher than ∼4.4 mL L^−1^ and 80% respectively, and Z_2 mL/L_ deeper than ∼40 m.

Mean cross-shore patterns of DO, DO_sat_ and Z_2 mL/L_ (Fig. 3B) also show evidence of decadal variations. The mean position by decade of DO, DO_sat_ and Z_2 mL/L_ isolines equal to 4.4 mL L^−1^, 80% and 40 m, respectively, is shown in Figure 3B. Highly-oxygenated surface waters (DO>4.4 mL L^−1^, DOsat>80%, Z_2 mL/L_>40 m) were located further than 55 km from the coast during the 1960s and moved progressively closer to the coast to reach minimum distances in the 1980s. After the 1980s the trends reversed and these waters retracted away from the coast during the 1990s (distances similar to the 1970s) and the 2000s but they have yet to reach the maximum values observed during the 1960s. These changes are associated with approaches and retreats of warmer, higher salinity (and higher oxygen) subtropical surface waters or inversely, the coastal expansion and contraction of the cooler, lower salinity coastal upwelling waters.

### Local scale

**Figure 4:**
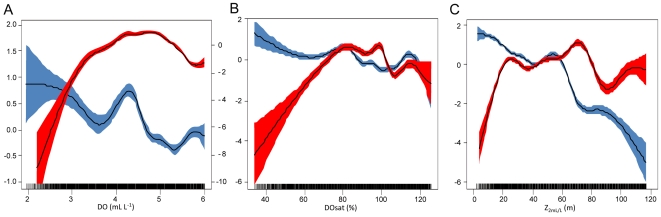
GAM models results. Cubic spline smoother fits (black solid lines) of GAMs (colored areas show 95% confidence limits) of anchovy (blue areas) and sardine (red areas) acoustic local biomass according to **A**. DO in mL L^−1^, **B**. DO_sat_ in % and **C**. Z_2 mL/L_ in m. The y-axes are relative and correspond to the spline smoother that was fitted to the data such that a y-value of zero is the mean effect of the variables on the response. Right y-axis corresponds to sardine in **A**.

General additive models (GAMs) show that DO, DO_sat_ and Z_2 mL/L_ were significantly correlated (*p = 0.000*) with fish biomass (Fig. 4). The effect of oxygen on anchovy biomass was weaker than for sardine; however anchovy was more abundant when DO, DO_sat_ and Z_2 mL/L_ were lower than ∼4.5 mL L^−1^, ∼85% and ∼40 m, respectively. Sardine show a strong negative effect of DO, DO_sat_ and Z_2 mL/L_, in particular when these decreased below ∼3.8 mL L^−1^, ∼60–70% and ∼25 m, respectively.

## Discussion

The results show that fish distribution and abundance in the coastal southeastern tropical Pacific are correlated to near-surface oxygen concentration/saturation and oxycline depth over a wide variety of scales. Further anchovy and sardine do not respond in a similar manner, anchovy shows an out-of-phase relationship compared to sardine. During the 1960s-early 1970s and the l990s–2000s, when DO and DO_sat_ were low and Z_2 mL/L_ shallow, anchovy was abundant but sardine did not proliferate. The opposite occurred during the late 1970s and 1980s when sardine exploded. It appears as if the turning point is when DO, DO_sat_ and Z_2 mL/L_ are greater or below ∼4 mL L^−1^, ∼70–80% and ∼25–35 m, respectively. Fish need oxygen to breathe, and getting oxygen inside their bodies is expensive energetically and more difficult at lower concentrations [30] but why such differences between species?

### Larger fish need more oxygen

Anchovy and sardine are gregarious fish and obligate ram ventilators. Oxygen supply per body size decreases as fish size/weight increases [30]. Sardine are larger fish than anchovy. Also the comparison of respiration rates between sardine and anchovy indicate a higher demand for sardine than for anchovy (*Engraulis capensis*) [39]. Finally [35], [40] showed that anchovy does not seem affected by a very shallow oxycline (<10 m) while sardine avoid such areas. Not only can the smaller anchovy survive in a very shallow oxycline but escapes predation by larger fish in doing so.

### Oxygen consumption, trophic structure and habitat compression

Oxygen demand also depends on the quality and quantity of food [30]. The Peruvian anchovy gets most of its energy by visual selection and direct biting on macrozooplankton [19]. Filter-feeding is very expensive metabolically relative to biting for anchovies [18], [39]. Zooplankton are very likely concentrated by a shallow oxycline [9] favoring anchovy bio-energetics. In sharp contrast, filter-feeding is energetically much cheaper for sardines [39], and the reason they might require more vertical habitat. These conclusions are supported by measurements of zooplankton size-structure which also exhibited multi-decadal fluctuations [41]. Euphausiids and large copepods were in-phase with anchovy peaks while sardine was more abundant when small zooplankton dominated [41]. During periods of high euphausiids and anchovy abundance the oxygenated vertical habitat was restricted to a very shallow layer concentrating the prey near the surface enhancing anchovy foraging (see [34]). Off Peru, ∼79% of the macrozooplankton, in particular euphausiids, perform vertical migration and distribute below the oxycline during the day [42]. During the night these organisms concentrate in the surface layer where they are foraged by anchovy, that adapt their foraging strategy (e.g. feeding time) to prey composition and concentration [19], [41]. The shallower the oxycline, the more concentrated the prey.

**Figure 5:**
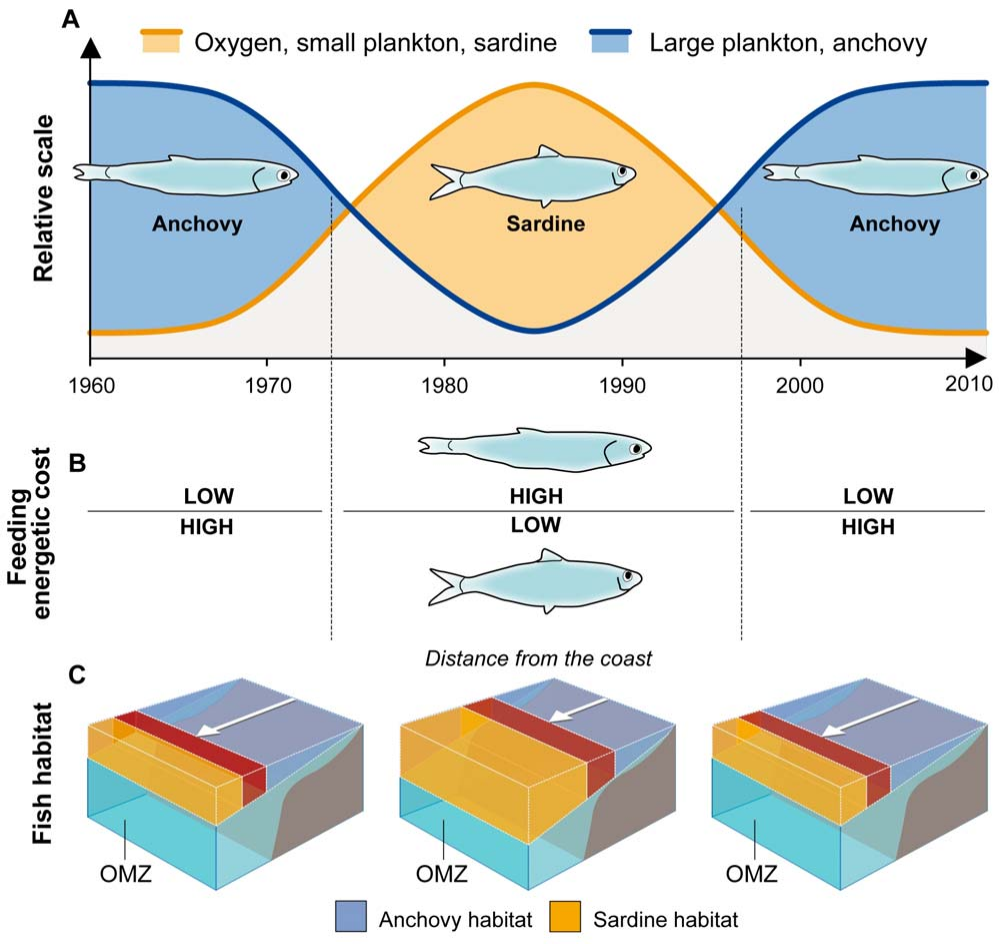
Conceptual model of decadal changes in anchovy and sardine populations in the southeastern tropical Pacific. **A**. Schematic of the temporal evolution of large plankton and anchovy (blue solid line), and oxygen, small plankton and sardine (red solid line) between 1960 and 2010. **B**. Energetic costs of feeding on dominant plankton size-spectra for anchovy and sardine according to the scenarios from **A**. **C**. Schematic of the available habitat for anchovy (blue shaded area) and sardine (red shaded area).

The results lead us to suggest the following conceptual model (Fig. 5). During the 1960s nutrient supply to the surface was enhanced by vertical displacement of isotherms upward, favoring large phytoplankton and zooplankton [43]. The vertical displacements reduced oxygen concentration and vertical habitat in the near-coastal regions, a condition that favored the anchovy which feeds on large zooplankton, tolerates low oxygen conditions and very narrow oxygenated habitats [35], [40]. In the early 1970s, climate changed, isotherms deepened and the system shifted toward less macrozooplankton [41], higher DO and a deeper oxycline. The anchovy populations dropped and sardine populations were favored. Anchovy collapse has been attributed to a combination of overfishing, an El Niño event and the decadal shift towards less productive conditions [14], [21]. The growth of sardines is linked here to a deeper and more oxygenated habitat and higher concentrations of small zooplankton that can be effectively harvested by filter feeding.

The 1980s were optimum for sardine: moderate productivity, greater concentrations of small zooplankton, more oxygen-saturated waters close to the coast, and a greater vertical oxygenated habitat. In contrast, conditions were less favorable for anchovy because of a reduction in nutrient supply, a narrow coastal habitat of upwelling/isotherm shoaling, increased predation, and a decrease in macrozooplankton [9], [21], [23], [41]. During the 1990s the climate reverted to conditions similar to the 1960s, early 1970s and led to a decrease/increase in sardine/anchovy populations. By the early 2000s the ecosystem was in a full anchovy regime [13], [22]. The system returned to one of high macrozooplankton abundance and dominance [41], [42], and low oxygen (both horizontally and vertically). The crash of the sardine population can be attributed to the synergetic effect of trophic structure and oxygen; these environmental effects were most likely aggravated by overfishing [11], [21], [27]. Prey was dominated by large zooplankton forcing sardine to shift toward a more energetically expensive direct biting, which became limiting due to changes in oxygen availability and vertical habitat. Sardine could not approach the coast and feed in the rich coastal habitat and slowly disappeared. Finally we can note that paleoecological studies show that sardine persistence was greater and variability reduced in Northern Chile relative to Peru [8]. We interpret this to be that oxygen is less regulating for sardines to the south. The OMZ is deeper off Chile [38] and the colder surface water can also contain more dissolved oxygen. In support of our findings it has been postulated that the sardine decline in the northern Benguela Current system was related to oxygen decrease in the spawning areas on the Namibian shelf [32], [44].

### Toward a 3D heuristic habitat-based hypothesis

The habitat-based hypothesis [21], [35], [40] suggests that spatial and temporal dynamics in habitat ultimately regulate fish distributions. For example, the anchovy habitat is restricted to the cold, upwelled coastal waters on a variety of scales [21], [23], [40]. Anchovy population dynamics has been shown to depend on the extension-contraction of these nutrient-rich waters, however, the habitat-based hypothesis did not account for why the sardine was restricted from nearshore waters and collapsed in the late 1990s. Oxygen adds the missing link and explains why sardine habitat moved further offshore where the retention of eggs and larvae is less efficient [45] and the quantity of food drops, weakening larval feeding success and survival. A large offshore low oxygen habitat is ideal for anchovy (Fig. 5C), allowing it access to the high concentrations of macrozooplankton at the shelf break [42]. Competition for food and predation on fish eggs, larvae and adults is reduced by the ‘expulsion’ of species that cannot survive in low oxygen conditions. Sardine benefit from a more oxygenated-deeper habitat (Fig. 5C). During these multi-decadal periods productivity is reduced but it is still high relative to other ecosystems and allows sardine accessing to coastal areas of enhanced productivity.

In conclusion, oxygen appears to provide the missing link needed to explain pelagic fish spatiotemporal dynamics and regimes in the southeastern tropical Pacific. Does habitat compression by oxygen also lead to the incredible production of fish observed off Peru relative to the other EBUS [9]? Is it because the prey are concentrated leading to an increase in fish carrying capacity or are the fish themselves concentrated making them more vulnerable to fishing? The former is more likely but these and other questions about oxygen and ecosystems remain and answers will be needed sooner rather than later given reports of expanding OMZs and eutrophication driven decreases in oxygen [5]–[6], [46]. How these changes will affect EBUS, which sustain ∼20% of worldwide fish harvests [47] remains uncertain.

## Materials and Methods

This study focuses on the coastal southeastern tropical Pacific between 7°S and 18°S. The area is characterized by low oxygen, cold and fresh water in the coastal surface layers and higher oxygen, warm and salty subtropical surface water further offshore [23], [38].

### Environmental data

An archive of ∼15 000 vertical profiles of temperature, salinity, and DO has been assembled for the region between 7°S and 18°S, an area where the OMZ is homogeneous [38], and from the Peruvian coast to 400 km offshore. Temperature and salinity were required to compute DO_sat_. These profiles were collected between 1961 and 2008 and obtained from the IMARPE and the World Ocean Database (WOD09, [48]). We have used both point-sampled stations (Nansen and Niskin bottles) and continuously sampled traces from conductivity–temperature–depth probes and profiling floats.

First, casts were interpolated onto 55 standard depth levels distributed between the surface and 1000 m depth, using an algorithm based on [49]. The vertical profiles do not properly identify “surface data” because the sample closest to the surface is in average located from 5–10 m depth. The near-surface reference depth was thus set at 10 m. The percentage saturation was calculated from DO_sat_ = (DO/DO′)×100, where DO′ is the solubility of oxygen at the *in situ* temperature and salinity measured at 10 m depth.

Second, for each interpolated profile, the oxycline depth was defined as the depth where the DO equals 2 mL L^−1^ (Z_2 mL/L_). A linear interpolation between standard levels is used to estimate the exact depth at which the value of 2 mL L^−1^ is reached. Note that [35] showed that the lower oxycline depth (or the top of the OMZ) corresponds to a DO of ∼0.8 mL L^−1^ (Z_0.8 mL/L_). This value was estimated taking into account all organisms (fish, zooplankton and gelatinous), but epipelagic fish, which are the focus of this study, are most often found when DO is higher than 1.5–2 mL L^−1^ (e.g. [32]–[33]). As a result we used a DO value of 2 mL L^−1^ as a proxy of the oxycline depth taking into account that the oxycline is sharp off Peru and that Z_2 mL/L_ and Z_0.8 mL/L_ are significantly correlated (p = 0.000, R^2^ = 0.91).

In order to study the mean large-scale interannual variations of oxygen parameters we focused on the stations located between the coast and 200 km offshore. From the ∼11 000 remaining values, we computed annual averages between 1961 and 2008. The annual time series were further smoothed using a non-parametric spline model with a degree of freedom (*df*) ranging from 4 to 5 (Fig. 1A–C) minimizing the GCV. Four-years moving average are also indicated in Figure 1A–C.

Cross-shore variations of oxygen properties were also investigated by computing mean profiles between the coast and 400 km offshore for each decade from the 1960s to 2000s. These mean cross-shore profiles were also smoothed using a non-parametric spline model (e.g. Fig. 3A). We determined the relative position from the coast of the 4.4 mL L^−1^, 80% and 40 m isolines from the filtered decadal cross-shore profiles (Fig. 3B).

Finally, to study fish-oxygen interactions at a local scale we used environmental information collected during acoustic surveys. DO and Z_2 mL/L_ data were interpolated using the natural neighbor method and averaged at the scale of acoustic data. Then corresponding data of salinity and temperature were used to calculate DO_sat_.

### Fish data

Anchovy and sardine landings (1964–2008) and biomass estimates from virtual population analysis (VPA) series (1964–2006) were provided by IMARPE. Acoustic fish biomass data were collected from 1983 to 2008 by IMARPE during 49 surveys performed on a variety of research vessels (see [22]). These surveys consisted of parallel cross-shore transects of ∼100 nm long, with a ∼15 nm spacing. Simrad (Kongsberg Maritime AS, Norway) scientific echosounders working at distinct frequencies were used to estimate biomass abundances (see [22]). Extensive midwater-trawl sampling accompanied the acoustic surveys for species identification and biological samples. Nautical-area-backscattering coefficients were recorded in each georeferenced elementary distance sampling unit (EDSU) equal to one or two nautical miles depending on the sample period. Identification of echoes was accomplished using metrics of the echoes and the catches from associated fishing trawls. Biomass estimates based on the acoustic backscatter for each species were carried out by IMARPE for each survey (see [22]).

### Data analysis

The effect of DO, DO_sat_ and/or Z_2 mL/L_ on pelagic fish was studied in three ways: (i) large temporal scale to study the decadal patterns between 1964 and 2008; (ii) cross-shore patterns; and (iii) small scale patterns (one EDSU) using data from the 44 acoustic surveys performed between 1983 and 2005. Different analyses were performed at each scale.

### Large scale

To remove high frequency variability (which is addressed at the local scale) and focus on decadal trends, annual time series of near-surface DO_sat_, Z_2 mL/L_, fish landings, and fish VPA and acoustic biomass estimates were smoothed using a non-parametric spline model (*df* ranged between 4 and 5). The resultant filtered time series were cross-correlated to ascertain whether the time series are in-phase, unrelated or alternate in phase. To test for the robustness of these cross correlations we performed them with the unsmoothed data and obtained similar results (Fig. S1). The cross-correlations significance levels were not corrected for possible biases due to temporal autocorrelation. Autocorrelation should reduce the degrees of freedom for the cross-correlation significance tests. However, the high cross-correlations for lags around 0 suggest that the series were either in or out of phase (Figs. 2 and S1). A very small p-value (p = 0.001) for significance was chosen (Fig. 2).

### Cross-shore profiles

In order to study the mean cross shore variations from the coast to 400 km offshore, DO, DO_sat_, Z_2 mL/L_, and acoustic fish biomass estimates were smoothed using a non-parametric spline model. Decadal changes in the cross-shore profiles of ambient parameters (DO, DO_sat_, and Z_2 mL/L_) were also examined by applying the same procedure on decadal datasets between the 1960s to the 2000s. For each decade the distance from shore where surface DO, DO_sat_ and Z_2 mL/L_ are equal to 4.4 mL L^−1^, 80% and 40 m, respectively was calculated.

### Local scale

At the EDSU scale, we used Generalized Additive Models (GAMs) to test the effect of DO, DO_sat_ and Z_2 mL/L_ on local fish acoustic biomass. Fish acoustic data are characterized by a large number of zeros (86% of our dataset). This feature generates a non-Gaussian distribution of the data which limits the use of GAMs. To avoid such limitation we modeled the GAMs applying the Tweedie versatile family distribution [50], which are commonly used to represent distributions that have a non-zero probability for the zero value.

## Supporting Information

Figure S1 **Time-lagged cross-correlations in year performed from unsmoothed data between anchovy and sardine catches, VPA-estimated and acoustic biomasses, and DO, DO_sat_ and Z_2 mL L_^−1^ at different time lags.** Values above (below, respectively) the top (bottom) dashed lines are significant at p = 0.01.
(TIF)
