# Correction: Oxygen: A Fundamental Property Regulating Pelagic Ecosystem Structure in the Coastal Southeastern Tropical Pacific

**DOI:** 10.1371/annotation/891b6bd0-185f-4f5f-9f0c-d2408a31f7f4

**Published:** 2012-02-21

**Authors:** Arnaud Bertrand, Alexis Chaigneau, Salvador Peraltilla, Jesus Ledesma, Michelle Graco, Florian Monetti, Francisco P. Chavez

There was an error in the funding statement. The correct funding statement is, “This work was supported by the cooperation agreement between the Peruvian Institute of the Sea (IMARPE) and the French Institute of Research for Development (IRD), of the International joint laboratory 'Dynamics of the Humboldt Current system' (LMI DISCOH) and of the project Peru Ecosystem Projection Scenarios (PEPS) funded by the French National Research Agency (ANR). Francisco P. Chavez was supported by the National Aeronautics and Space Administration (NASA) and the David and Lucile Packard Foundation. The funders had no role in study design, data collection and analysis, decision to publish, or preparation of the manuscript.” 

