# Supplementary figures and images for: Oxygen: A Fundamental Property Regulating Pelagic Ecosystem Structure in the Coastal Southeastern Tropical Pacific
Source: PLoS One. 2011 Dec 28;6(12):e29558. doi: 10.1371/journal.pone.0029558 (PMC3247266; doi:10.1371/journal.pone.0029558)

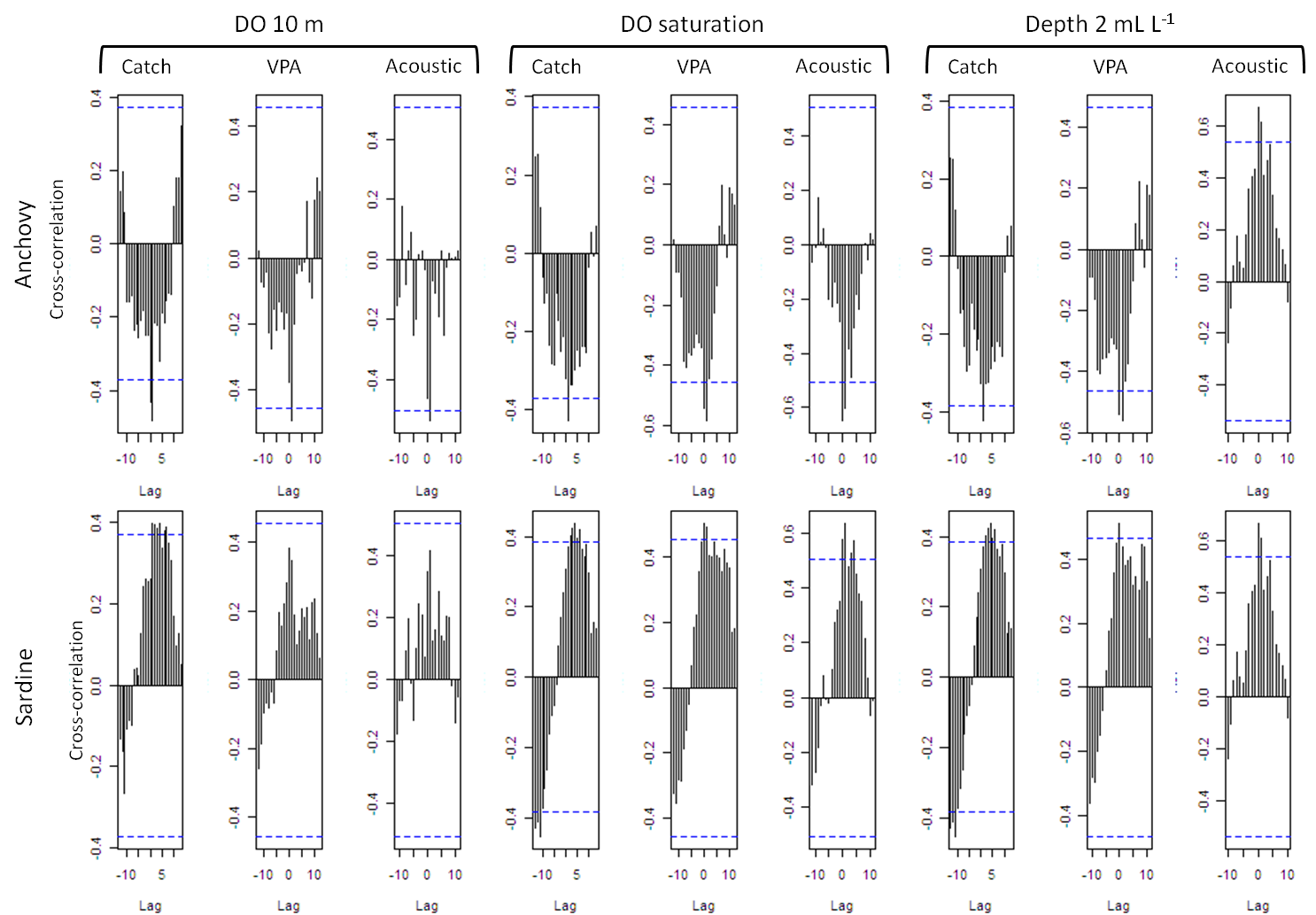

Supplement: Figure S1 — Time-lagged cross-correlations in year performed from unsmoothed data between anchovy and sardine catches, VPA-estimated and acoustic biomasses, and DO, DOsat and Z2 mL L−1 at different time lags. Values above (below, respectively) the top (bottom) dashed lines are significant at p = 0.01. (TIF) [file pone.0029558.s001.tif]
